# The Environmental Conditions, Treatments, and Exposures Ontology (ECTO): connecting toxicology and exposure to human health and beyond

**DOI:** 10.1186/s13326-023-00283-x

**Published:** 2023-02-24

**Authors:** Lauren E. Chan, Anne E. Thessen, William D. Duncan, Nicolas Matentzoglu, Charles Schmitt, Cynthia J. Grondin, Nicole Vasilevsky, Julie A. McMurry, Peter N. Robinson, Christopher J. Mungall, Melissa A. Haendel

**Affiliations:** 1 Oregon State University, Corvallis, OR 97331 USA; 2 University of Colorado Anschutz Medical Campus, Aurora, CO 80054 USA; 3 University of Florida, Gainesville, FL 32610 USA; 4 Semanticly, Athens, Greece; 5 National Institute of Environmental Health Sciences, Durham, NC 27709 USA; 6 North Carolina State University, Raleigh, NC 27965 USA; 7 The Jackson Laboratory for Genomic Medicine, Farmington, CT 06032 USA; 8 Lawrence Berkeley National Laboratory, Berkeley, CA 94720 USA

**Keywords:** Biomedical ontology, Environmental exposures, Environmental health

## Abstract

**Background:**

Evaluating the impact of environmental exposures on organism health is a key goal of modern biomedicine and is critically important in an age of greater pollution and chemicals in our environment. Environmental health utilizes many different research methods and generates a variety of data types. However, to date, no comprehensive database represents the full spectrum of environmental health data. Due to a lack of interoperability between databases, tools for integrating these resources are needed. In this manuscript we present the Environmental Conditions, Treatments, and Exposures Ontology (ECTO), a species-agnostic ontology focused on exposure events that occur as a result of natural and experimental processes, such as diet, work, or research activities. ECTO is intended for use in harmonizing environmental health data resources to support cross-study integration and inference for mechanism discovery.

**Methods and findings:**

ECTO is an ontology designed for describing organismal exposures such as toxicological research, environmental variables, dietary features, and patient-reported data from surveys. ECTO utilizes the base model established within the Exposure Ontology (ExO). ECTO is developed using a combination of manual curation and Dead Simple OWL Design Patterns (DOSDP), and contains over 2700 environmental exposure terms, and incorporates chemical and environmental ontologies. ECTO is an Open Biological and Biomedical Ontology (OBO) Foundry ontology that is designed for interoperability, reuse, and axiomatization with other ontologies. ECTO terms have been utilized in axioms within the Mondo Disease Ontology to represent diseases caused or influenced by environmental factors, as well as for survey encoding for the Personalized Environment and Genes Study (PEGS).

**Conclusions:**

We constructed ECTO to meet Open Biological and Biomedical Ontology (OBO) Foundry principles to increase translation opportunities between environmental health and other areas of biology. ECTO has a growing community of contributors consisting of toxicologists, public health epidemiologists, and health care providers to provide the necessary expertise for areas that have been identified previously as gaps.

**Supplementary Information:**

The online version contains supplementary material available at 10.1186/s13326-023-00283-x.

## Introduction

Environmental health is a branch of public health that encompasses the study of the inter-relationship between organisms (typically humans) and environmental conditions that may impact their health. Environmental health includes investigations into toxic exposures, but it can also encompass exposure to chemicals and environments such as vitamins, climate, and social stressors. Identification of stimuli in environmental substances is critical for disease prevention and management of adverse health outcomes, as well as to identify and evaluate mechanisms of action to develop clinical treatments. Environmental health has evolved alongside other fields including genomics, phenomics, nutrition, epidemiology, and crop sciences. Each of these interconnected disciplines are essential to understanding the full picture of how environments can prevent, cause, or ameliorate disease.

Toxicology is an important sub-field of environmental health. Existing toxicology-focused databases and data repositories such as Chemical Effects in Biological Systems (CEBS) [1], Comparative Toxicogenomics Database (CTD) [2], National Health and Nutrition Examination Survey (NHANES), and Aggregated Computational Toxicology Resource (ACToR) databases [3], currently house a mix of structured, semi-structured, and unstructured information regarding environmental exposure impacts on a variety of species [4]. These resources offer unique features, including being repositories for raw data from toxicology studies, aggregating and inferring findings from the literature, or housing survey questions and results. For some of these data resources (e.g., NHANES surveys) Common Data Elements (CDEs) are utilized, which include standardized survey questions and responses intended to unify data from multiple resources using the same CDEs. While the attempts to align a variety of related but heterogeneous data resources using CDEs are meaningful, unfortunately, CDEs are often lacking in their computational encoding, making them challenging to use for making data interoperable [5].

Resources such as the Human Health Exposure Analysis Resource (HHEAR) [6], the Unified Medical Language System (UMLS) [7] and the Adverse Outcomes Pathway Knowledgebase (AOP) [8] utilize existing ontology terminology in their modeling (e.g. chemical entities from the Chemical Entities of Biological Interest Ontology, ChEBI). However, content regarding environmental exposures is still needed within AOP and UMLS, and analytical opportunities and widespread uptake are still limited using HHEAR.

Even the most comprehensive resources are still limited by their lack of standardized language, computational structure, or cross-study and cross-discipline data comparison capabilities. In efforts to support data integration within and beyond environmental health, a common standard for describing and coordinating these data is necessary.

Currently, ontologies related to environmental exposure are limited, with most ontologies focused on the description of environments, chemicals, or species-specific exposure conditions. However, no species-agnostic exposure ontology that includes the stimuli and media currently exists. This limits researchers’ ability to represent exposure events in a standardized way when working with unrepresented model organisms. The lack of a unifying exposure ontology hinders the harmonizations of existing and future data regarding environmental exposures and related health outcomes.

A demand for integration of environmental health into interoperable data resources using ontologies is documented [9–11], with a variety of toxicologists, public health epidemiologists, and health care clinicians seeking established standards and resources. For this reason, we have created the Environmental Conditions, Treatments, and Exposures Ontology (ECTO) to satisfy the gaps seen within current ontology resources and to provide a translation tool for toxicology and biological data integration. ECTO’s exposure event structure is a species-agnostic approach that can be used to align existing environmental health databases and resources. For example, CTD offers highly relevant data regarding exposure stimuli including potential biological ramifications of exposure and references to literature. While it is meaningful data, the data is structured in a format that does not create context for the exposure itself (e.g., multiple rows of data may relate to an exposure to chlorpyrifos and list some reported outcomes, but the outcomes are not coordinated with each other to provide an exposure phenotype profile or to compare to any known diseases and their common phenotypes). By utilizing the computable structure of ECTO, resources like CTD could be directly aligned with other ECTO compatible resources and could be leveraged for inference regarding exposures and human phenotype or disease outcomes across data sources. Additionally, ECTO follows Findable, Accessible, Interoperable, and Reusable (FAIR) principles [12].

## The Environmental Conditions, Treatments, and Exposures Ontology (ECTO)

ECTO contains compositional classes which utilize content from other biomedical ontologies (such as the Environment Ontology (ENVO) [13] and the Chemical Entities of Biological Interest (ChEBI) [14]) to create exposure classes. Examples of exposures represented in ECTO include experimental treatments and interventions used in research (e.g. toxicological investigations), exposures experienced by humans or other organisms in daily life, natural and artificial stimuli experienced by organisms, and environmental conditions or ecosystems experienced by a single organism or population of organisms. By maintaining a general scope of terms, ECTO can provide a wide range of content that can be applied in research settings ranging from wet lab to clinical care. Included in ECTO’s exposure content are an organism’s internal and external exposures, mixtures of known and inferred exposures, and indication of the route and medium of exposure when available.

**Fig. 1:**
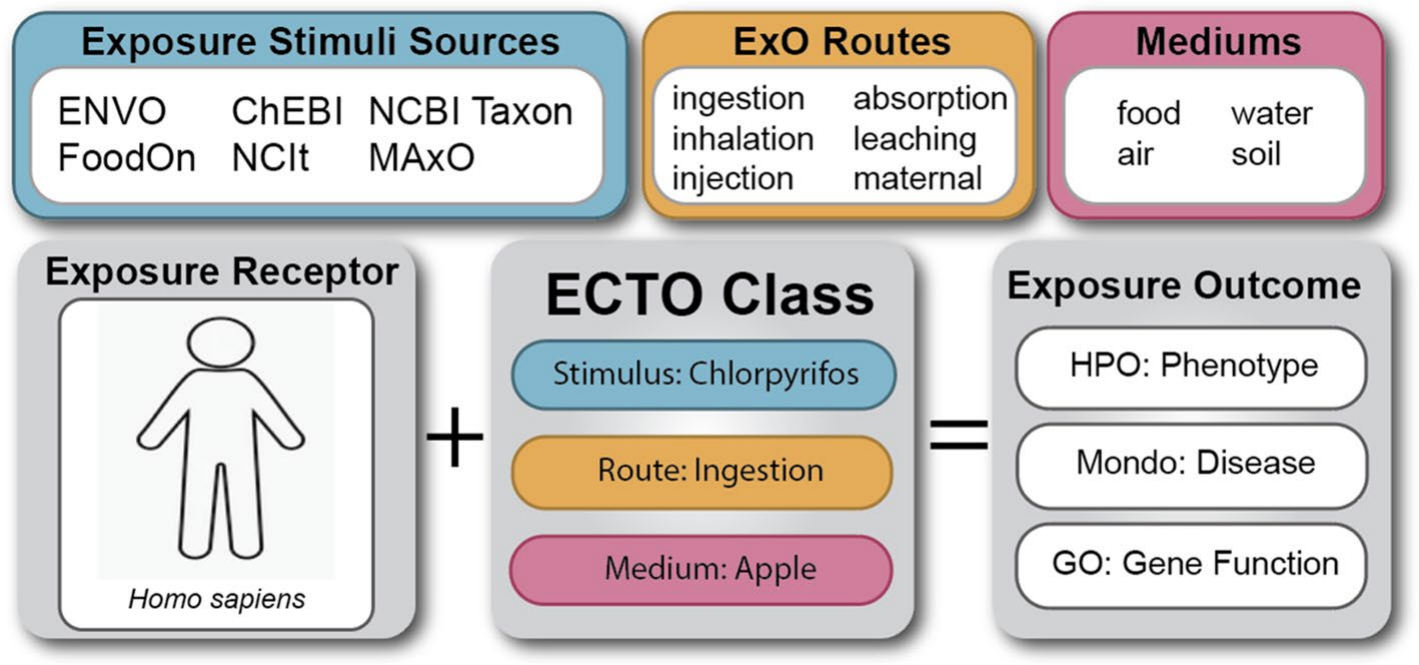
ECTO unifies exposure attributes. Existing ontologies contain terms describing common exposure stimuli, exposure routes, and potential exposure media, however unifying terms to describe the exposure process were not yet represented. Utilizing the schema of the Exposure Ontology (ExO), ECTO classes can coordinate the stimulus, route, and exposure components into a single process term for consistent exposure classes. Logical axioms can be used to define the relationships between exposure receptors, ECTO classes, and documented exposure outcomes from the literature. Instance level schemas can also be developed using individual data points as the exposure receptor or exposure outcomes. Abbreviations: ENVO, The Environment Ontology; ChEBI, Chemical Entities of Biological Interest; NCBI Taxon, National Center for Biotechnology Information Taxonomy; FoodOn, Food Ontology; NCIt, National Cancer Institute Thesaurus; MAxO, Medical Actions Ontology; HPO, Human Phenotype Ontology; Mondo, Mondo Disease Ontology; GO, Gene Ontology

For example, acute and chronic dietary exposure to agricultural chemicals may pose a risk to human health, particularly for children and developing fetuses [15]. Chlorpyrifos was banned for household use in the US in 2000, but up until recently it has continued to be used in American agriculture, regardless of potential detrimental health effects [16]. Figure 1 showcases ECTO’s unification capacity, coordinating existing ontologies or data sources as part of an ‘exposure to chlorpyrifos’, which even in low doses may have resulting phenotypes such as a runny nose, tears, or drooling [17]. In this example, a person presented to a health care provider complaining of an ongoing runny nose, tears, and drooling in recent history with no known illness, and they report eating apples daily. Our existing knowledge from our exposure event structure includes 1) chlorpyrifos is sprayed on some apples, and 2) runny nose, tears, and drooling are all chlorpyrifos associated phenotypes. Having structured knowledge in a format that supports queries, we can more quickly identify the exposure concern and provide an intervention. This structure would also allow for alignment of heterogeneous databases and data sources such as electronic health record and survey-based resources.

The primary audience for ECTO includes toxicologists, clinicians, integrative and/or computational biologists, and exposure researchers who are seeking a standard for documenting environment and exposure-based interventions. Additionally, ECTO is intended to serve environmental epidemiologists whose experimental designs may focus on identifying environmental exposures impacting their subjects. In turn, researchers who are interested in any related areas of biology can then also capitalize on any indicated relationships between organism exposure and health outcomes. A variety of competency questions and use cases have been documented by stakeholders including toxicologists, public health epidemiologists, and clinicians [9]. Some examples can be seen in Supplement Table 1.

## ECTO’s methodological framework

ECTO is available on GitHub [18]. Many aspects of the ECTO life cycle, in particular, release workflows, continuous quality control testing and management of ontology dependencies (imports) are delivered using the Ontology Development Kit (ODK) [19]. The development of ECTO, in particular the definition of new terms, is driven largely by Dead Simple OWL Design Patterns (DOSDPs). The basic idea is to define logical patterns which are populated with terms from external ontologies (such as CHEBI and ENVO) to generate logical axioms for new terms. These axioms can then be used by an automated reasoner to classify exposures according to the classifications provided by the external ontologies.

**Fig. 2:**
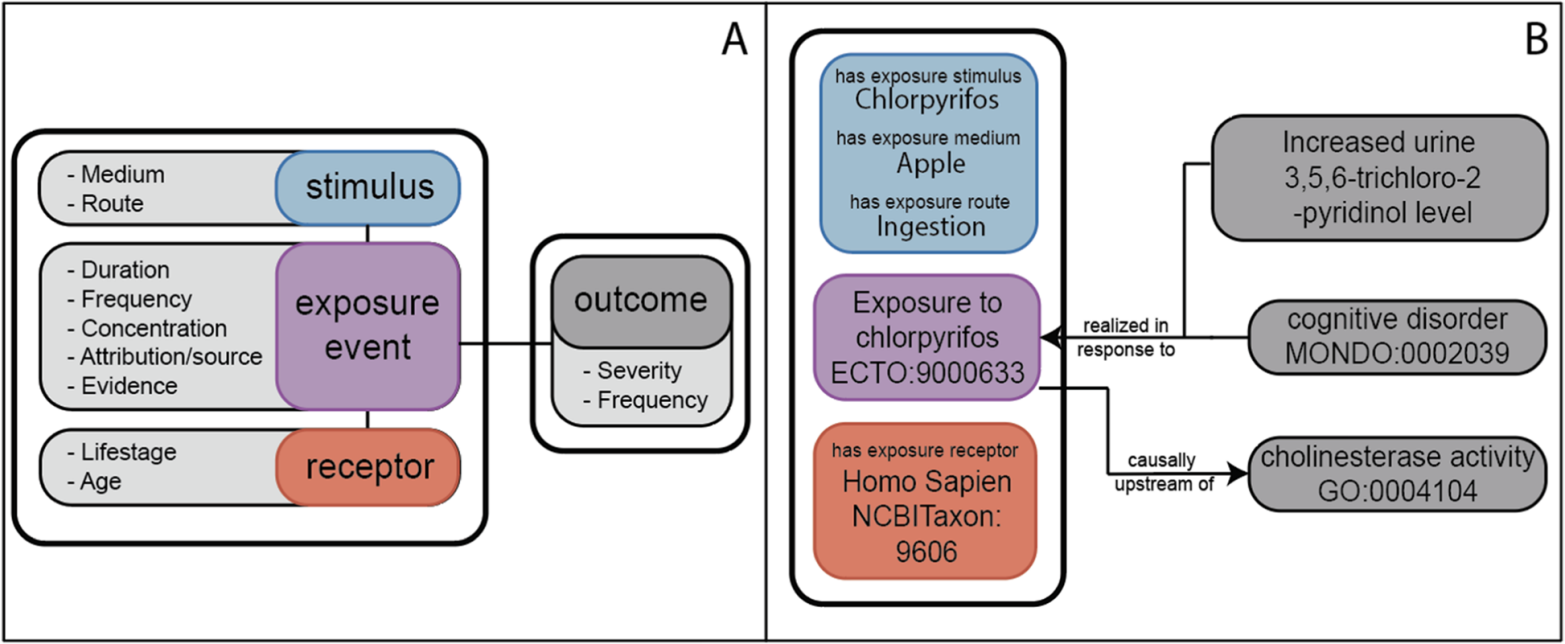
Overview of exposure schema. **A** ExO Upper Level Schema: Modified from Thessen et al. [9], the ExO schema for modeling exposure events forms the base infrastructure for ECTO. **B** ECTO exposure schema. The colors in panel **B** reflect the superclasses in panel **A** (e.g., stimulus is blue, exposure event is purple, and receptor is red, outcomes are in dark gray). Utilizing the ExO schema, ECTO terms include detailed information regarding the stimulus, medium, and route of an exposure. Relationships from the OBO Relations Ontology also facilitate annotations of the exposure receptor and a variety of exposure outcomes. Further annotations regarding data specific information such as temporality or dose of the exposure can be included as annotations within a knowledge graph or other computational data structure

Exposures modeled in ECTO are based on the upper level Exposure Ontology (ExO) [20], offering specific content such as an exposure to a chemical, an environmental condition, or a mixture of components. Created by exposure science community researchers in 2012, ExO contains a high level toxicology schema to connect exposure stimuli, receptors, routes, and media as described in Fig. 2A. The ExO schema describes the components of an environmental exposure event, however it does not contain any classes referencing specific types of exposures (e.g. exposure to lead) and merely provides the structured aspects of an exposure. Leveraging this structure, we have developed ECTO to host classes that are templated based on the ExO model, that include information about the stimulus, and in some cases the medium or route of exposure. Relationships between ExO classes, and subsequently ECTO classes (e.g. realized in response to) are all sourced from the Relations Ontology [21]. The contextual aspects of the exposure including temporality and location are currently modeled as annotations in the ECTO model and are encouraged to be included as components of postcomposed classes or as annotations. We are currently working on requirements for best practices and documentation for use of ECTO in curated annotations akin to a Gene Ontology Association File (GAF) [22] that will include post composition guidelines. Figure 2B showcases the detailed modeling that is achievable using ECTO exposure terms as well as the logical axioms that can be instantiated based on literature findings.

ECTO treats exposures as events; in ontological terms, they are types of occurrents (e.g., an entity with temporal parts and that happens, unfolds or develops through time). As a subclass of occurrent, the exposure event includes interactions between a receptor (typically an organism, but could be a population of organisms or an organism part) and a stimulus (an agent or process that has a potential effect on the receptor). The stimulus may interact with the organism through some kind of environmental medium (e.g., air, water, soil), and may enter via some route (e.g., permeating the skin or analogous barrier). In turn, the exposure terms in ECTO range from somewhat broad terms (e.g., exposure to lead) to more specific (e.g., exposure to lead in water via ingestion). ECTO terms follow a standardized nomenclature of ‘exposure to X’ with ‘X’ referring to an ‘exposure stimulus’ term that is an existing ontology term, and the ability to add variable terms referring to the medium and route if required. By utilizing terms from existing ontologies, ECTO can additionally harmonize content from other databases annotated to the terms (e.g., CAS Registry Numbers for chemical terms). Inclusion of annotations like temporality of exposure or dose of exposure may be desired within a data model for analysis. Annotation models, analogous to those used for the Gene Ontology [23], can be used to annotate ECTO terms within a knowledge graph format to associate instance level data with the standardized exposure terms. While many potential exposure terms may be developed (e.g., exposures for all chemical entities in ChEBI), we intend to utilize precomposition for terms that are likely to be reused by a variety of users. We are focused on precomposing exposure terms that are indicated for a specific use case, or that have documented effects in the literature. For more specific exposures, we plan to leverage our developing annotation model to support use cases, without inflating the ontology.

## Content creation for ECTO

**Fig. 3:**
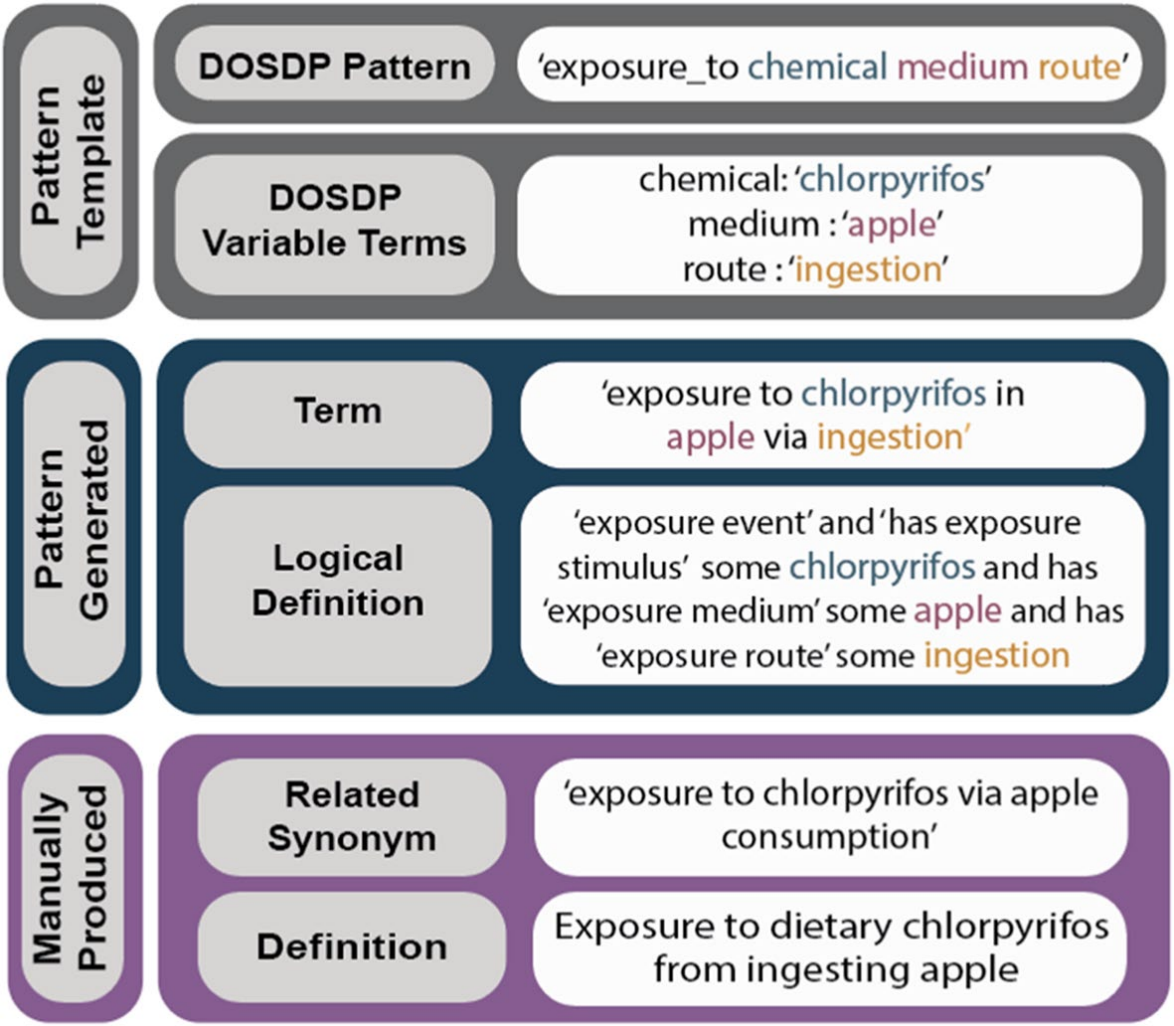
Chlorpyrifos Exposure in Apple Design Pattern. Represented is the ‘exposure to chemical medium route’ DOSDP, which can be used to create the term ‘exposure to chlorpyrifos in apple via ingestion’ and its logical definition using existing ‘chlorpyrifos’, ‘apple’ and ‘ingestion’ terms and the DOSDP template developed by a curator. Synonyms and human readable definitions can be manually added to the term or can also be included within the DOSDP if appropriate

Terms are developed for ECTO using both expert manual curation using the Protege ontology editor tool [24] and pattern-based curation. To avoid ECTO becoming overly complex and subsequent maintenance challenges, a pattern-based annotation format can be used to describe unique features of the stimulus, receptor, exposure event, and outcome. Pattern-based curation is conducted using Dead Simple OWL Design Patterns (DOSDPs) [25]. DOSDPs are easy to read, YAML based templates for generation of ontology content including labels, synonyms, text definitions, and logical axioms. DOSDPs are particularly useful in their ability to reference existing terms from other ontologies, which for ECTO is an essential component to term development. DOSDPs contain set classes and relationships that can be used to structure logical axioms, as well as variable fields which will differ for each class created with the template. Application of DOSDPs is further depicted in Fig. 3.

Three distinct axiomatic patterns have been developed specifically for use within ECTO, including Exposure, Exposure + Route, and Exposure + Route + Medium pattern formats. Current patterns in ECTO can be viewed on GitHub [26].

**Fig. 4:**
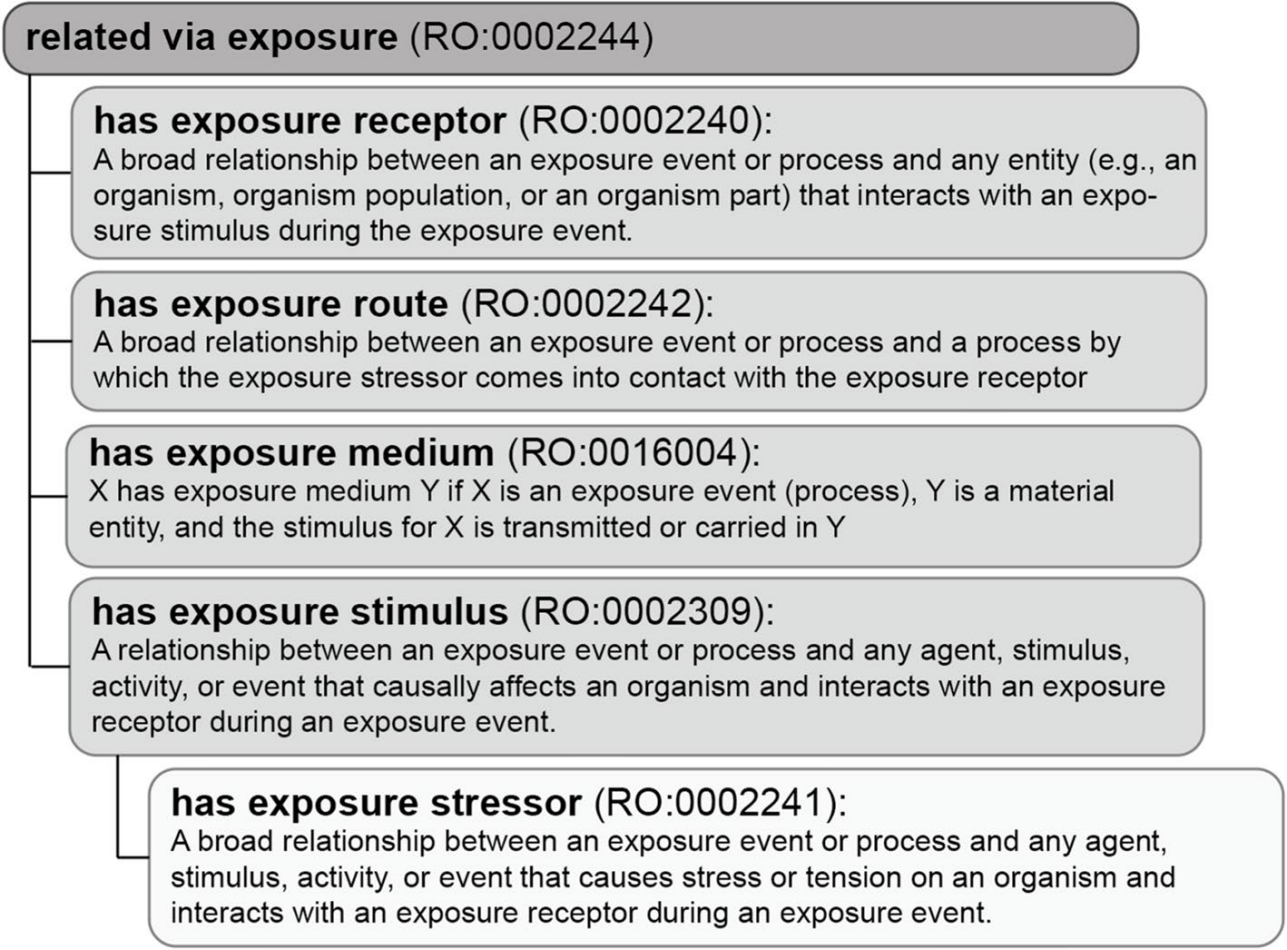
Exposure Based Relations. The OBO Relations Ontology contains a variety of relationship terms that fall under the superclass of ‘related via exposure’. Each of these relations can be used in conjunction with ECTO terms to create an exposure schema

In both precomposed ECTO terms and in postcomposed annotations, the Relation Ontology (RO) provides standardized relationships, including those seen in Fig. 4.

## Using ECTO in data annotations

Annotations using ECTO are created by associating environmental exposures to a phenotype, disease, gene, or behavior in efforts to create a depiction of current exposure knowledge. Annotations can include a variety of information such as temporality, concentration/dose, and related evidence that connects the exposure to the term of interest.

ECTO annotations contain the following components:

- ECTO term (required)
- Associated phenotype/disease/behavior/gene (required)
- Reference (required if assertion is from literature)
- Evidence (required)

ECTO annotations are intended to be supported either directly or indirectly by relevant and accurate scientific literature. Information from databases and resources such as CTD or TOXNET can also be leveraged for annotations. For example, with the use of ECTO terms, annotations from CTD can be integrated into the larger Monarch knowledge graph [27]. Similarly, data from the National Toxicology Program (NTP) [28] could be structured using ECTO in combination with dosing and timing regimens along with outcomes encoded using uPheno or HPO as an annotation file format for use in downstream computation.

ECTO terms are used to axiomatize exposure-related diseases in the Mondo Disease Ontology (Mondo) [29]. Mondo integrates several underlying disease terminologies and ontologies into a merged resource that provides semantic mappings to source ontologies [30]. Mondo provides a library of DOSDPs, including a pattern for diseases where the cause of the disease is an exposure to an environmental stimulus [31]. Axiomizating Mondo using these standard exposure patterns allows for auto-classification of the hierarchy, and an overall more robust description of the disease term. This pattern is now used for 46 different exposure influenced disease terms in Mondo.

## ECTO and model organism research

Exposure modeling and annotations can be particularly useful for toxicology research using model organisms. Robust phenotype ontologies have been developed for model organisms, such as the Zebrafish Model Organism Network (ZFIN) which describes genetic, genomic, phenotypic, and developmental data for zebrafish [32], and the overarching Unified Phenotype Ontology (uPheno) which integrates multiple phenotype ontologies into a unified cross-species phenotype ontology [33].

Ontologies or standards for experimental conditions exist for some model organisms. For example, the Zebrafish Experimental Conditions Ontology (ZECO) describes experimental designs in zebrafish studies [34]. Planteome, a network of ontologies that integrate data from experiments on plants, offers a Plant Trait Ontology (TO) as well as a Plant Experimental Conditions Ontology (PECO) [35]. PECO terms describe common treatments, growing conditions, and/or study types used in plant biology experiments. Similarly to how uPheno has been developed for the unification of cross-species phenotype content, we hope ECTO can follow a similar approach to offer cross-species content regarding environmental conditions and treatments for any model organism or humans.

Modeling goals and development strategies (e.g., DOSDP) are aligned for ECTO as well as ZECO and other specific environmental condition ontologies. The similar construction offers the opportunity for a higher-level unification (such as seen within phenotype ontologies and uPheno) through OWL Axiomatization and OWL Reasoning. If all experimental condition ontologies document the semantic axioms within their individual content, a reasoner (such as ELK or HermiT) can evaluate multiple ontology terms and find the overarching classes being referenced.

For example, the PECO term ‘formaldehyde exposure’ contains the logical axiom:

- plant exposure and *has exposure stimulus* some formaldehyde

And a related ECTO term ‘exposure to formaldehyde’ has the logical axiom:

- exposure event and *has exposure stimulus* some formaldehyde

These two similar logical axioms contain the same relationship of ‘has exposure stimulus’ and the stimulus of ‘formaldehyde’, so while the exposures (plant exposure vs exposure event) may differ, their logical axioms still allow for adequate association of the terms and a link between two related but distinct exposures. For example, while plant exposure is currently not formally defined as an exposure event which pertains to plants, if such a definition would be added a reasoner could determine ‘formaldehyde exposure’ in PECO is a subclass of ‘exposure to formaldehyde’ in ECTO. (Note that it is not good practice across OBO ontologies to use the same labels for different concepts (exposures to plants vs general exposure), but it is general practice in species specific ontologies such as anatomy, phenotype, and experimental conditions to not include species information in labels.) Additional attention should be provided to taxon restrictions, term definitions, logical axioms, and the ontology a term is found in to ensure appropriate usage.

While ECTO has similar modeling structures to related ontologies, ECTO is distinct in its descriptions of species agnostic exposures to environmental entities, chemicals, and other stimuli. Distinctions between ECTO and related ontologies are described in Supplemental Table 2.

## Use cases

### PEGS use case

An initial use case for ECTO was provided by the National Institute of Environmental Health Sciences (NIEHS) and their Personalized Environment and Genes (PEGS) research [36]. PEGS researchers are focused on the variety of ways in which environmental exposures impact organism health. This use case is intended to identify methods for parsing environmental exposure and health data collected via self-reported survey and evaluate associations between singular or combined exposures that are associated with an adverse health outcome.

**Fig. 5:**
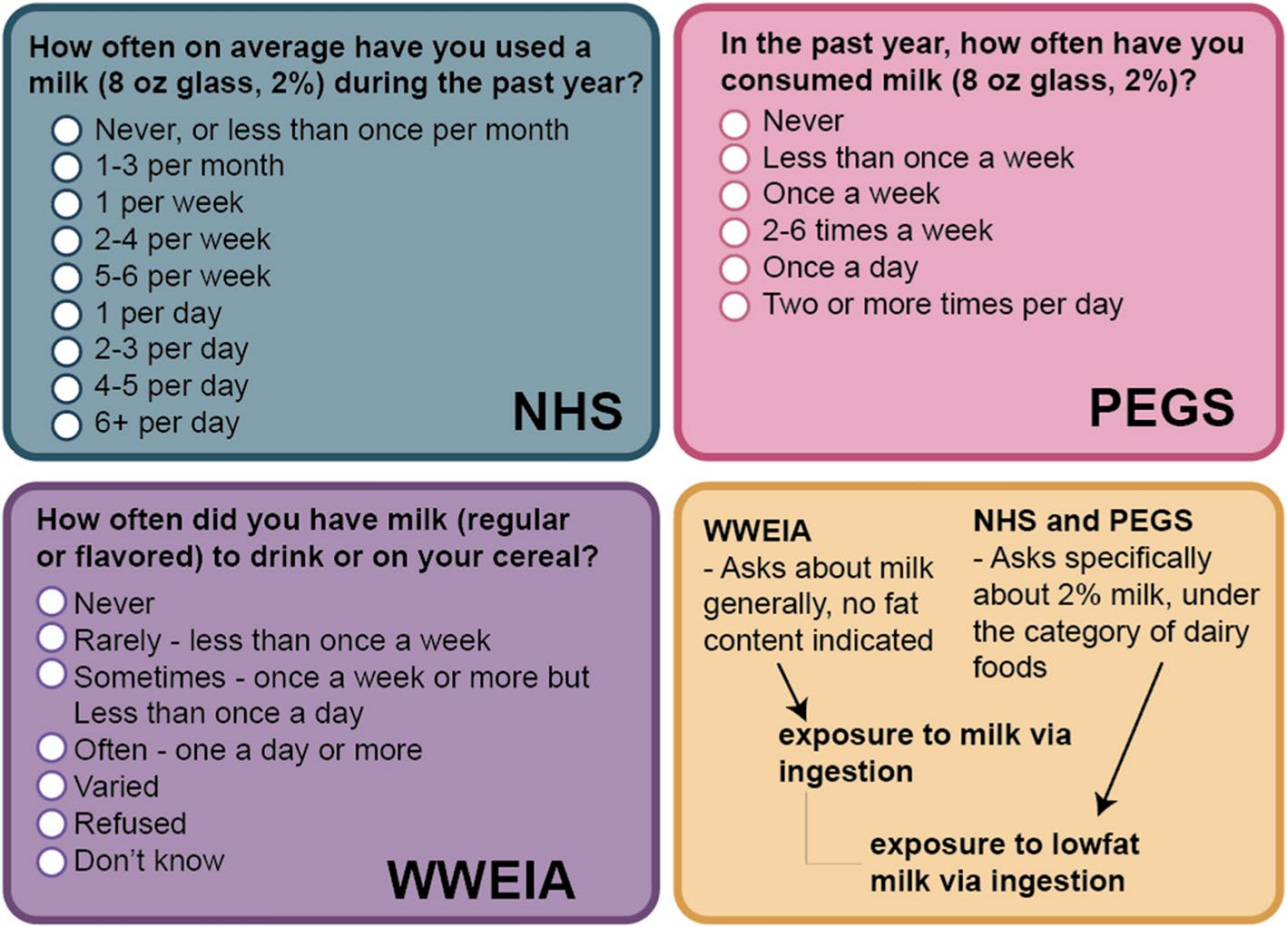
Making Common Data Elements (CDEs) interoperable. Surveys can include question and response CDEs. A variety of CDE registries exist, but not all of them are interoperable and some CDEs may be duplicative in their content. Three different surveys and CDEs can be seen in blue, purple, and green, which compare similar questions about milk from the Nurses’ Health Study (NHS), What We Eat in America (WWEIA) and the PEGS surveys. While each question asks about milk, the responses elicited from each are not directly compatible and can be difficult to computationally assess. An ontology centric approach assesses each question and the resulting responses for the common exposure feature which can be classified using the ontology hierarchy and annotated in a knowledge graph to encompass a variety of potential responses for harmonization

PEGS has developed three surveys for self-reported data collection including Health and Exposures, Internal Exposome, and External Exposome surveys. Each survey was developed to include some CDEs, however as previously noted CDEs are limited in their computational capacity and often inadequately aligned across survey tools for integrated data analysis. Our use case focused on ontological encoding of each survey question using ECTO to provide standardized language and computational structure to each survey item (Fig. 5). This approach utilized hand curation to identify variables of interest for each survey item, and corresponding ontology classes. In turn this methodology is a template for mapping preexisting data from heterogeneous surveys to align and compare findings. Additionally, these methods will support future survey development to enhance immediate data interoperability.

The content of the PEGS surveys was utilized as a primary resource for common workplace, home, hobby, and activity-based exposures that informed ECTO’s initial precomposed exposure classes.

**Fig. 6:**
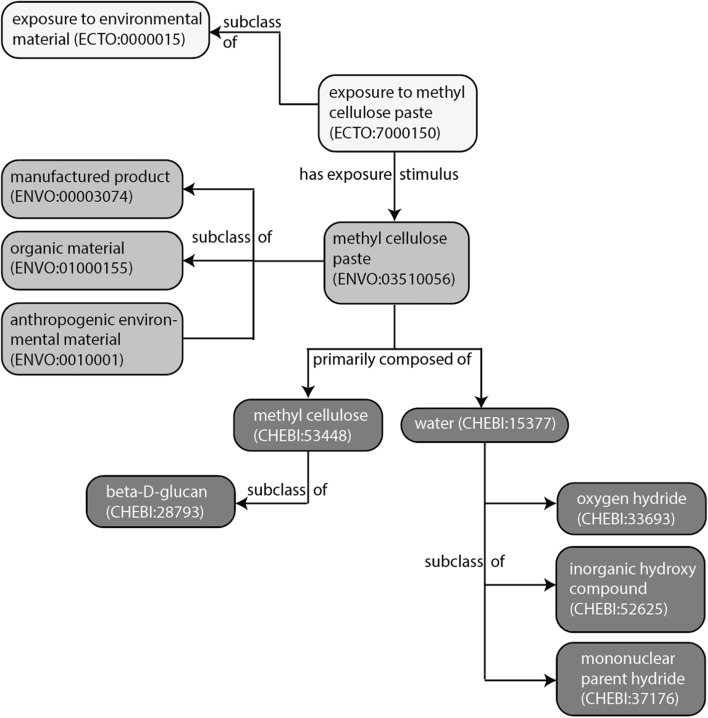
Relating ontologies with ECTO. Mixture exposures in ECTO must have the stimuli represented as its own class in another ontology (e.g., ENVO) for ECTO to reference in the exposure class. Here, the ECTO class ‘exposure to methyl cellulose paste’ and its parent are in white, its stimulus ‘methyl cellulose paste’ and the related ENVO hierarchy are in light gray, and the subcomponents of the mixture, ‘methyl cellulose’ and ‘water’ are seen in dark gray, along with their ChEBI hierarchies

Of particular interest to PEGS researchers was the creation of mixture exposure terms that include metadata regarding each component of the mixture. To create mixture terms, we worked with the ENVO team to template all necessary elements for each term including information about the mixture components (e.g., methyl cellulose paste is composed of methyl cellulose and water, with the axioms ‘*composed primarily of*some water’ and ‘*composed primarily of*some methyl cellulose’). Then we created the subsequent exposure term using the axiomatized ENVO class (e.g., exposure to methyl cellulose paste). The axiomatic relationships between ECTO and other ontologies are visualized in Fig. 6.

Using this structure, the survey data can be classified based on exposure to the mixtures or exposure to the components within the mixture. Additionally, the exposure terms can also be classified based on the inherent relationships within the ontology (e.g., an exposure to sulfuric acid can be classified with other exposures to acids), further supporting higher powered assessment. Development of precomposed exposure terms for this particular use case was driven by a one-to-one mapping method of ECTO exposure terms to PEGS survey questions currently being used to develop a knowledge graph for data visualization and querying. While this project is still ongoing, the precomposed exposure terms have facilitated straightforward mapping techniques. Additionally, this project has used precomposed terms primarily as the previously proposed work including ExO’s exposure schema has gone largely underutilized even while it was developed with many stakeholders from the exposure community. With precomposed terms, because of the axiomatization contained within each term we are able to quickly and efficiently query using SPARQL to identify exposure event classes even when no specific named class exists. An example query to identify all exposure classes that include a stimulus that is a subclass of cow milk (liquid) (FOODON:03302116) can be seen below:

Endpoint: https://ubergraph.apps.renci.org/sparql

- PREFIX owl: <http://www.w3.org/2002/07/owl#>
- PREFIX obo: <http://purl.obolibrary.org/obo>
- PREFIX rdf: <http://www.w3.org/1999/02/22-rdf-syntax-ns#>
- PREFIX rdfs: <http://www.w3.org/2000/01/rdf-schema#>
- PREFIX FOODON: <http://purl.obolibrary.org/obo/FOODON_>
- SELECT? exposure? label_exposure? stimulus? label_stimulus WHERE {
- ?exposure <http://purl.obolibrary.org/obo/RO_0002309>? stimulus .
- ?exposure rdfs:label? label_exposure .
- ?stimulus rdfs:subClassOf* FOODON:03302116 .
- ?stimulus rdfs:label? label_stimulus .
- }

### Zebrafish use case

**Fig. 7:**
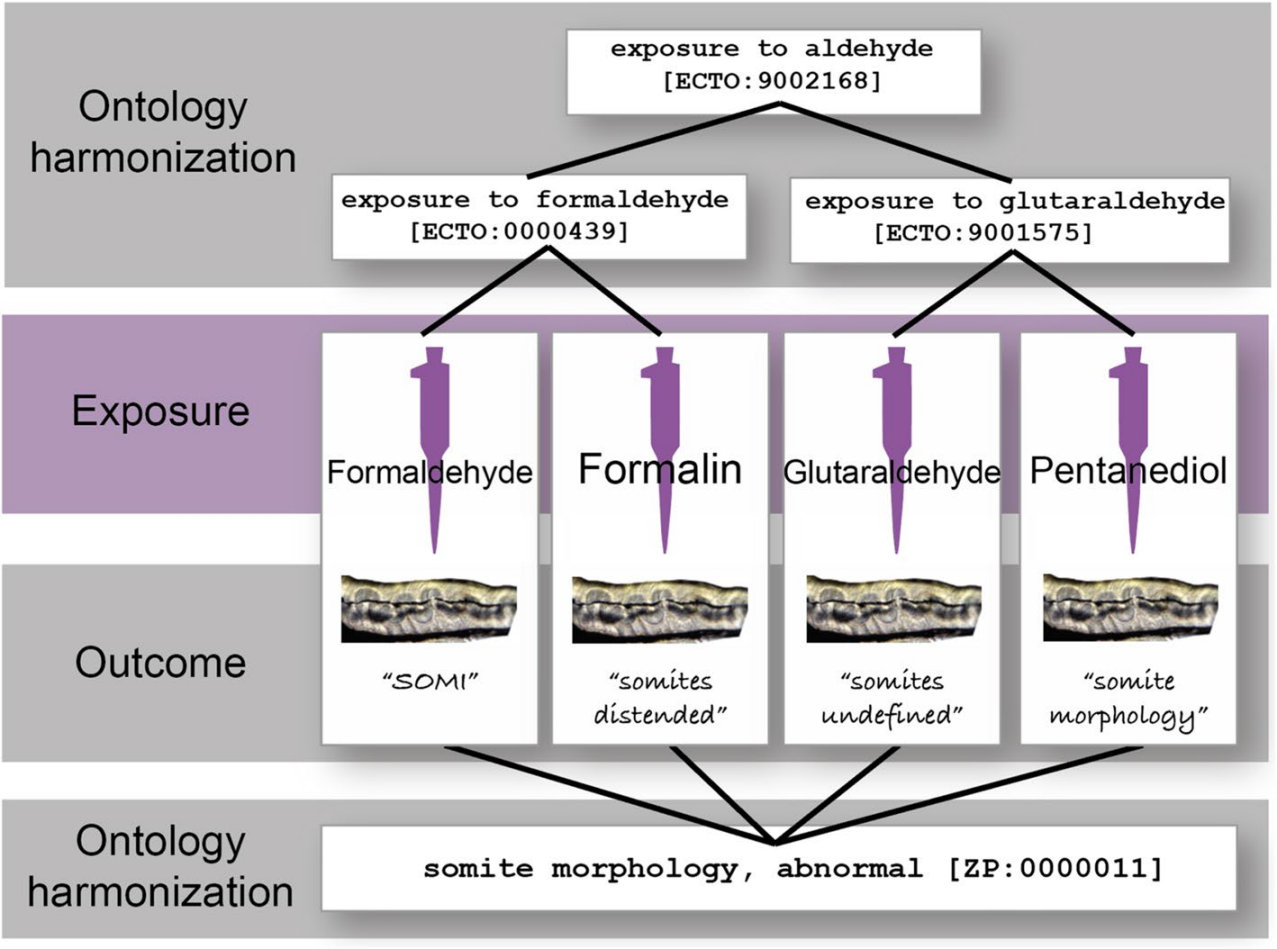
Standardizing exposures and outcomes in zebrafish. In this study, zebrafish embryos were exposed to an aldehyde by four different labs (formaldehyde CHEBI:16842 and glutaraldehyde CHEBI:64276). Once hatched, the zebrafish were observed for irregular phenotypes, and all displayed an abnormality of their somites. The names used to describe the stimulus and the outcome was different in each of the four labs. Using ontologies like the Zebrafish Phenotype Ontology (ZP), the Environmental Conditions, Treatments, and Exposures Ontology (ECTO), and the Chemicals of Biological Interest Ontology (ChEBI) we can integrate data from four different labs even though they use different terms and different stimuli

We have also used ECTO for the annotation of toxicology studies, such as exposure investigations in zebrafish (*Danio rerio*). Zebrafish are a commonly used toxicological model organism due to a variety of features such as low cost, quick breeding cycle, and transparent embryos [37]. However, it has been challenging to compare results of studies performed in different laboratories because the way in which the exposure chemicals, methods and parameters, and resulting phenotypes are encoded is laboratory specific. Further, in some cases the chemicals themselves are obfuscated due to partnerships with commercial entities. However, it is still possible to classify such chemicals into higher level categories such as “exposure to aldehydes”. Without the computational mappings of ontology terms and logical axioms to this instance level data, researchers would have to integrate manually. By enriching these data with ontology terms, we have empowered researchers to efficiently integrate heterogeneous data across labs at scale for more powerful statistical and meta-analyses (Fig. 7).

## Limitations of ECTO

ECTO currently describes a wide array of chemical and natural or built environmental exposures, but it does not yet include exposures to infectious agents, many foods, nutrients, social environments (e.g., education, crime, and access), as well as more unique or complex multi-layered exposures (e.g., exposure to UV radiation while wearing SPF 30 sunscreen).

Another limitation of ECTO is its reliance on existing ontology content for the development of exposure terms. While many stimuli are represented in robust ontologies like ChEBI and ENVO, our team is consistently pursuing content requests in other ontologies to create ECTO terms for our use cases. This reliance can complicate ECTO workflows in terms of release delays, reasoning errors, or other potential issues from ingested ontologies that can be introduced into ECTO.

## Conclusions

ECTO is a computational ontology designed to support any type of exposure event to any type of organism. It can be utilized to harmonize data from across sources and data modalities, such as surveys, literature annotations, toxicological studies, and in clinical research. ECTO will continue developing content for exposures to allergens, foods and nutrients, hobby and occupational exposures, and geographic location-based exposures. We are particularly interested in coordinating dietary survey information from specific geographical regions with agricultural chemical usage data to ask questions such as “If a person ate an apple grown in Washington, are they likely to be exposed to chlorpyrifos?”, “what if the apple is washed?”, “was it an organic apple?” and other layers of questions to infer dietary exposures. We hope that by asking questions such as these within knowledge graphs and other instance level data visualizations, we can infer what and how exposures may be occurring, potentially assert some general quantification information on the exposure, and if available coordinate exposure findings with documented health outcomes in the respondent. With continued development of ECTO and its use in logical axioms like in Mondo disease ontology, we plan to integrate environmental exposures and coordinated health outcomes into the diagnostic tools The Monarch Initiative currently supports.

This manuscript introduces the Environmental Conditions, Treatments, and Exposures Ontology (ECTO) as described using the minimum information for the reporting of an ontology (MIRO) guidelines [38].

Ontology Owner: The Monarch Initiative.

Contact: Anne Thessen, annethessen@gmail.com

License: CC BY 3.0.

Ontology URL: http://www.obofoundry.org/ontology/ecto.html

Ontology Repository: https://github.com/EnvironmentOntology/environmental-exposure-ontology

We welcome user requests for new terms and other contributions via our issue tracker (https://github.com/EnvironmentOntology/environmental-exposure-ontology/issues).

## Supplementary Information

**Additional file 1: Supplement Table 1.** ECTO Applications.

**Additional file 2: Supplemental Table 2.** Comparison of ECTO scope to other ontologies [39–42].

## Data Availability

Not applicable.
