# Supplementary material for: The Environmental Conditions, Treatments, and Exposures Ontology (ECTO): connecting toxicology and exposure to human health and beyond
Source: J Biomed Semantics. 2023 Feb 24;14:3. doi: 10.1186/s13326-023-00283-x (PMC9951428; doi:10.1186/s13326-023-00283-x)
Supplement: Supplementary file 1 — Additional file 1: Supplement Table 1. ECTO Applications. [file 13326_2023_283_MOESM1_ESM.pdf]

Supplement Table 1. ECTO Applications

	Bench Science/Translation	Clinical Research	Epidemiology/Ecosystem
**General Use Case**	Standardizing descriptions for toxicological experimental methods to offer coordination of data from same model studies as well as from different/higher level model studies for translational science.	Describing standardized exposures occurring in humans in coordination with clinical health outcomes, including genotype, phenotype, and disease.	Establishing structured concepts for single or composite exposures impacting large populations of humans or ecosystem environments.
**Example Specific Use Case**	Translating toxicological endpoint terms, like neurodevelopmental toxicity, into phenotype terms from HPO (Human Phenotype Ontology)	Coordinating an individual's exposure to air pollutants at their home and workplace with their clinical phenotypes and health outcomes.	Predict what a disease cluster would look like based on the structure and/or composition of a chemical or mixture that is similar to the chemical/mixture in question.
**Example Competency Questions**	What genetic variants are associated with greater sensitivity to altered neurologic function from exposure to polychlorinated biphenyls?<br><br>What are the developmental effects in animals and humans exposed prenatally to benzene over short or long periods of time? What are the methylation tag pattern changes related to prenatal exposure to benzene in animals?	What levels of benzene exposure in the air over what period of time are likely to cause blood cancers? Which blood cancers?<br><br>What is my biggest exposure risk based on my geographical location? How can I prevent or minimize that exposure? What am I exposed to in my particular line of work? How might this impact my health?	Which chemicals in stormwater run-off have aquatic toxicity data available from assays done at different salinities?<br><br>What is the effectiveness of environmental regulations for reducing health impacts from air and water contaminants? What sources of chemical exposures exist in my community?

A variety of use cases have been identified for ECTO based on previous workshops with stakeholders in this field. We foresee three primary theme areas in which ECTO may be utilized (stated in the column headers) and provide examples of what each theme entails.
