# Supplementary material for: The Environmental Conditions, Treatments, and Exposures Ontology (ECTO): connecting toxicology and exposure to human health and beyond
Source: J Biomed Semantics. 2023 Feb 24;14:3. doi: 10.1186/s13326-023-00283-x (PMC9951428; doi:10.1186/s13326-023-00283-x)
Supplement: Supplementary file 2 — Additional file 2: Supplemental Table 2. Comparison of ECTO scope to other ontologies [39–42]. [file 13326_2023_283_MOESM2_ESM.pdf]

Supplemental Table 2. Comparison of ECTO scope to other ontologies

Ontology	Status	No. of Classes	Scope	Description	URL or Reference
Environmental Conditions, Treatments, and Exposures Ontology	Active	2763	Exposures to environments, substances, behaviors, and scenarios.	A structured vocabulary for experimental, clinical, and natural world exposures.	https://github.com/EnvironmentOntology/environmental-exposure-ontology

Ontology	Status	No. of Class references in ECTO	Scope	Description	URL or Reference
Environment Ontology (ENVO)	Active	333	Environmental features and habitats	Concise, controlled description of environments.	[39]<br><br>https://github.com/EnvironmentOntology/envo
Exposure Science Ontology (ExO)	Active	57	Higher level environmental structure	Vocabularies for describing exposure data	[20]<br><br>https://github.com

				to inform understanding of environmental health.	/CTDbase/exposure-ontology
Chemical Entities of Biological Interest (ChEBI)	Active	2170	Chemicals, molecular entities	A structured classification of molecular entities of biological interest focusing on 'small' chemical compounds.	[14] https://github.com/ebi-chebi/ChEBI
Food Ontology (FoodOn)	Active	169	Food material, food processing	A controlled vocabulary of food products and entities bearing a “food role”	[40] https://github.com/FoodOntology/foodon
National Cancer Institute	Active	48	Broad coverage of the cancer	A thesaurus of cancer related diseases,	[41]

Thesaurus (NCIT)			domain	findings and abnormalities	
Experimental Conditions Ontology (XCO)	Active	0	Internal and external environmental conditions	A vocabulary of conditions under which physiological and morphological measurements are made.	https://jbiomedsem.biomedcentral.com/articles/10.1186/2041-1480-4-26<br><br>http://www.obofoundry.org/ontology/xco.html
National Center for Biotechnology Information (NCBI) Organismal Classification (NCBI taxon)	Active	11	Organism taxonomy	A classification and nomenclature for all of the organisms in the public sequence databases.	[42]
Ecocore	Active	0	ecological entities, such as ecological functions (for	Concise and controlled description of ecological traits	https://github.com/EcologicalSemantics/ecocore

			predators, prey, etc), food webs, and ecological interactions.	of organisms.	
Zebrafish Experimental Conditions Ontology (ZECO)	Active	0	Experimental conditions for zebrafish	Descriptions of experimental conditions applied to zebrafish.	[34] https://github.com/ybradford/zebrafish-experimental-conditions-ontology
Plant Experimental Conditions Ontology (PECO)	Active	0	Experimental conditions for plants	Descriptions of experimental conditions applied to plants.	[35] https://github.com/Planteome/plant-experimental-conditions-ontology

The ontologies and resources indicated within this table are utilized as imports within ECTO to support its precomposed exposure classes. In addition to the listed ontologies, ECTO also imports Gene Ontology, Information Artifact Ontology (IAO), Medical Actions Ontology (MAxO),

Neuro Behavior Ontology (NBO), NanoParticle Ontology (NPO), Phenotype and Trait Ontology (PATO), Relation Ontology (RO), and Uber-anatomy Ontology (UBERON).
